# Supplementary material for: Canagliflozin Attenuates Lipotoxicity in Cardiomyocytes by Inhibiting Inflammation and Ferroptosis through Activating AMPK Pathway
Source: Int J Mol Sci. 2023 Jan 3;24(1):858. doi: 10.3390/ijms24010858 (PMC9821072; doi:10.3390/ijms24010858)
Supplement: Supplementary file 1 [file ijms-24-00858-s001.zip › ijms-2082807-supplementary.pdf]

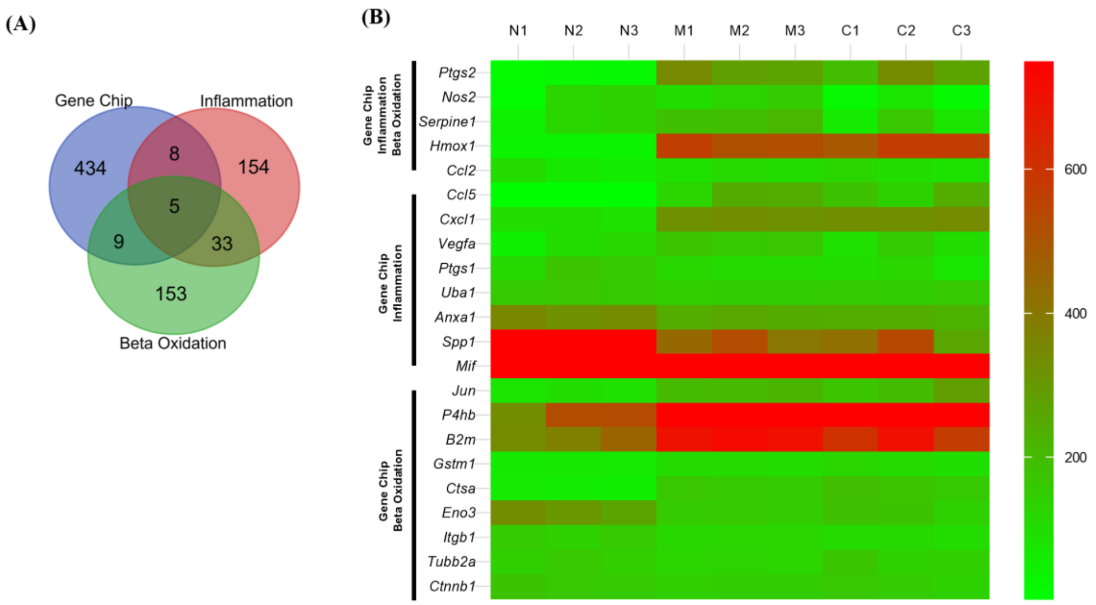

Figure S1. Canagliflozin regulates the expression of genes related to inflammation and beta oxidation in PA-treated HL-1 cells in gene chips. HL-1 cells were stimulated with 0.1mM PA for 12 h in the presence or absence of CAN (5 μg/mL). **(A)** 456 genes highly expressed in the gene chip (FPKM>100) were intersected with the top 200 genes of inflammation and beta oxidation in the GeneCards database, respectively, by Draw Venn Diagram, then the venn diagram of related genes in gene chips and GeneCards database was analyzed. **(B)** Heat map of 22 highly expressed genes in gene chips. Nor (N1, N2, N3), normal control group; Mod (M1, M2, M3), untreated PA control group; CAN (C1, C2, C3), CAN-treated PA group.

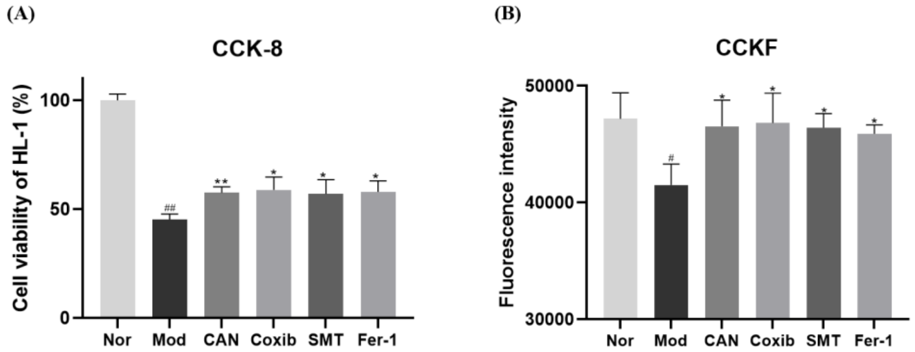

Figure S2. Effects of CAN, Coxib, SMT and Fer-1 on cell viability of HL-1 cells. HL-1 cells were stimulated with 0.1mM PA for 24 h. **(A)** Effects of CAN (5 μg/mL), Coxib (5 μg/mL), SMT (10 μM) and Fer-1 (5 μM) on cell viability of HL-1 cells, which was analyzed by Cell Counting Kit-8. **(B)** Fluorescence intensity of CAN, Coxib, SMT and Fer-1, which was analyzed by Cell Fluorescence Counting Kit. Nor, normal control group; Mod, untreated PA control group; CAN, CAN-treated PA group; Coxib, Coxib-treated PA group; SMT, SMT-treated PA group; Fer-1, Fer1-treated PA group. Data were expressed as mean ± SD (n = 3). Differences were considered significant at $P < 0.05$, and highly significant at $P < 0.01$ (#$P < 0.05$, ## $P < 0.01$ vs. Nor; * $P < 0.05$, **$P < 0.01$ vs. Mod).
